# Efficacy of concurrent chemoradiotherapy with retrograde super selective intra-arterial infusion combined with cetuximab for synchronous multifocal oral squamous cell carcinomas

**DOI:** 10.1186/s13014-023-02282-9

**Published:** 2023-05-26

**Authors:** Xuefei Chen, Mitomu Kioi, Yuichiro Hayashi, Toshiyuki Koizumi, Izumi Koike, Shoji Yamanaka, Masaharu Hata, Kenji Mitsudo

**Affiliations:** 1 Department of Oral and Maxillofacial Surgery, Yokohama City University Graduate School of Medicine, 3-9 Fukuura, Kanazawa-Ku, Yokohama, Kanagawa 236-0004 Japan; 2 Department of Radiation Oncology, Yokohama City University Graduate School of Medicine, Yokohama, Japan; 3 Department of Pathology, Yokohama City University Hospital, Yokohama, Japan

**Keywords:** Intra-arterial chemotherapy, Cetuximab, Oral squamous cell carcinoma, Synchronous multifocal cancer

## Abstract

**Background:**

The incidence of multicentric oral cancer is increasing. However, treatment encounters difficulty if each tumor needs to be treated simultaneously. The objective of this clinical case report is to highlight the effect of concurrent chemoradiotherapy with retrograde superselective intra-arterial infusion combined with systemic administration of cetuximab on synchronous multifocal oral squamous cell carcinomas.

**Case presentation:**

A 70-year-old man presented to the hospital with multiple tumors and oral pain. Three independent tumors were found in the right dorsal tongue, left edge of the tongue, and left lower lip. Based on the characteristic appearance of the lesions and further evaluation, clinical diagnoses of right tongue cancer “T3”, left tongue cancer “T2” and lower left lip cancer “T1”, N2cM0 were made. Treatment was initiated with systemic administration of cetuximab, followed by intra-arterial chemoradiotherapy. Treatment results were complete response on all three local lesions, and left neck dissection was performed following the initial treatment. The patient showed no evidence of recurrence during the 4 years follow-up period.

**Conclusions:**

This novel combination treatment seems to be a promising strategy for patients with synchronous multifocal oral squamous cell carcinoma.

## Background

Oral cancer represents approximately 2% of all human cancers and is the seventh most common cancer overall in the world [1, 2]. Along with extended life expectancy, the incidence of multicentric oral cancer is increasing, and hence treatment will be prolonged if each lesion is treated separately [3]. Surgical resection is the standard treatment for oral cancer patients, albeit the patient’s quality of life may significantly decline when patients have several tumors or advanced cancers in the oral cavity [4]. With respect to preserving oral function, concurrent chemoradiotherapy with retrograde superselective intra-arterial infusion (IACRT) plays an important role in treating locally advanced oral cancer. Better safety results and comparable efficacy of IACRT for oral cancer have been reported previously [5, 6]. However, it is not clear if the IACRT is effective for the treatment of patients with synchronous multifocal oral cancer, as those diseases might be required infusion of chemo-drugs via multiple routes, leading to reduction of efficacy and expanded area of mucositis.

Molecular-targeted drug, cetuximab, is currently utilized for the treatment of locally advanced and recurrent/metastatic head and neck squamous cell carcinoma in worldwide [7]. It is an anti-epidermal growth factor receptor monoclonal antibody, which can competitively inhibit the binding of epidermal growth factor (EGF) and tumor growth factor (TGF) alpha and can induce antibody-dependent cellular cytotoxicity (ADCC), reducing cell growth and metastatic spread [8]. In recent years, the combination of cetuximab with either radiotherapy (RT) or chemotherapy is commonly used. In locally advanced cases, cetuximab combined with RT is an alternative to radical treatment if the patient is unavailable to use the platinum drug.

In this article, we report a case of synchronous multifocal oral squamous cell carcinomas treated with IACRT and systemic administration of cetuximab with some review of the literature to estimate the efficacy and benefit of the treatment.

## Case presentation

**Fig. 1:**
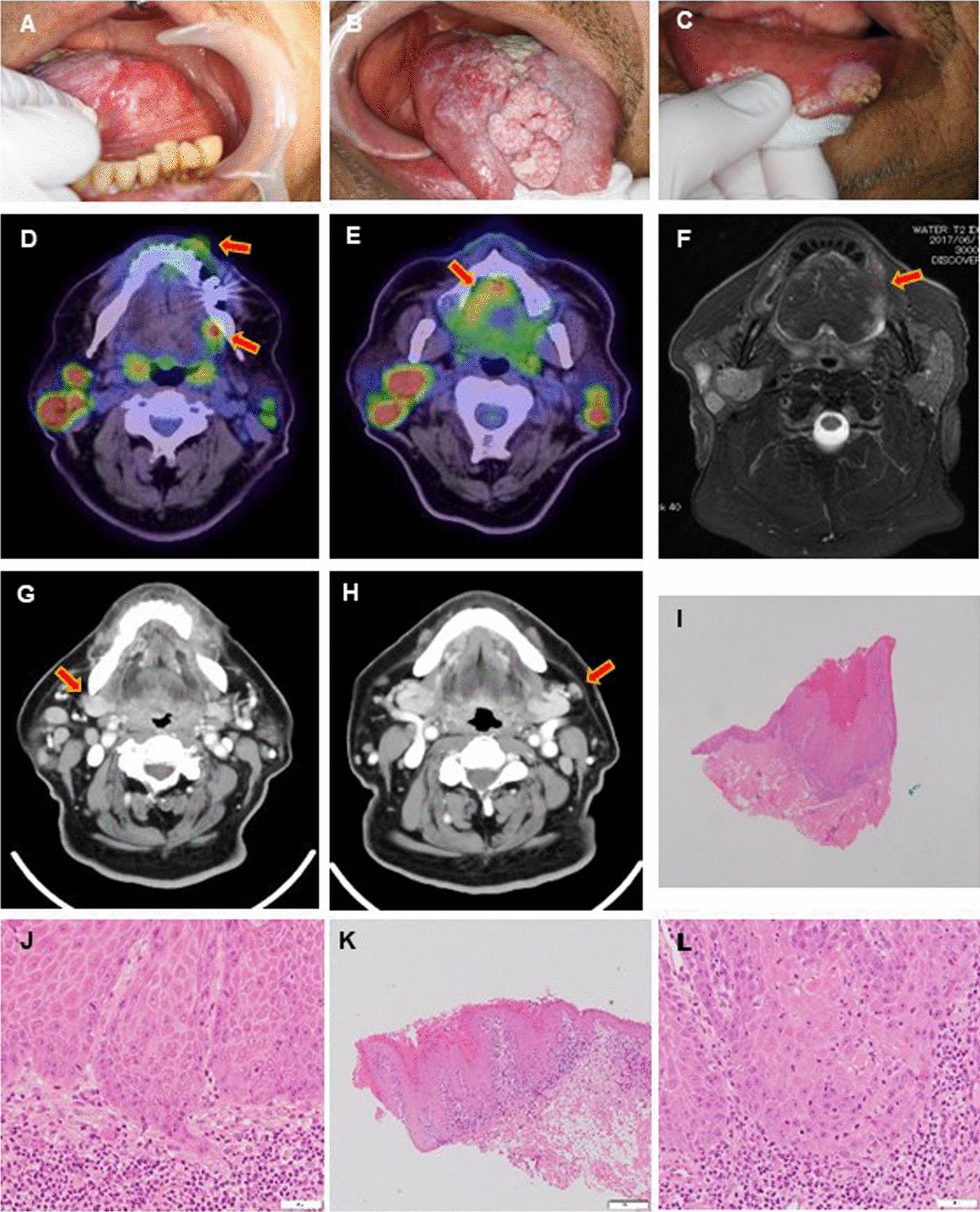
Representative images and pathology of synchronous multifocal oral squamous cell carcinomas. Three independent tumors were found in left tongue (**A**), right dorsal tongue (**B**), and left lower lip (**C**). **D**, **E** PET/CT image showed abnormal uptake of FDG (arrows, SUVmax: tongue = 8–11, lip = 6.2). Multiple abnormal accumulations also showed in the bilateral parotid glands at the same time. **F** Contrast T1-weighted image showed a mass with high signal intensity on the left margin of tongue (arrow). **G**, **H** CECT images showed the high density presented in right level IIA and left level IIB lymph node (arrows). **I**–**L** Microinvasion and cancer pearl, epithelial funiculus dysplasia and atypical mitotic figure can be seen in histopathology of the tumors (**I**, **J** right tongue; **K**, **L**, lip). Scale bars indicate 1 mm (**I**), 500 μm (**K**), and 50 μm (**J**, **L**)

A 70-year-old man with masses on his tongue and lower lip was referred to our hospital. He had no significant past history unless hypertension. On initial examination, the patient had three independent masses with induration at the right dorsum tongue, left lateral edge of the tongue, and left lower lip. Each tumor showed different characteristics (a 53 × 45 × 10 mm pedunculated tumor on his right dorsum of tongue, a 27 × 24 × 8 mm introverted tumor on the left lateral edge of tongue, and a 14 × 13 × 6 mm pedunculated tumor with partial keratinization on his lower lip) (Fig. 1A–C). 18-Fluorodeoxyglucose positron emission tomography/computed tomography (FDG-PET/CT) images revealed abnormal uptake of FDG (SUVmax = 6.2–11) by each legion of mass and bilateral cervical lymph nodes. Besides, multiple abnormal accumulations showed in the bilateral parotid glands (Fig. 1D, E), which were diagnosed with Warthin’s tumor and a follow-up examination was performed at the Department of Otolaryngology in our hospital. Contrast-enhanced MR image (T1-weighted) showed a 23 × 20 × 9 mm mass with high signal intensity on the left margin of tongue, and the tumor of right tongue and lip were unclear (Fig. 1F). Contrast-enhanced computed tomography (CE-CT) images showed the high density presented in level IIA at the right side and level IIB at the left side (Fig. 1G, H). The masses at right dorsum tongue, left side edge of tongue, and lower lip were diagnosed as well-differentiated squamous cell carcinoma (SCC) on pathologic examination of biopsy specimens (Fig. 1I–L). The distance between the tumor at the right dorsal tongue and that at the left side edge of the tongue was 34 mm. According to the 8th Edition of the UICC TNM classification of oral cavity cancers, the patient was definitively diagnosed with right tongue cancer “T3”, left tongue cancer “T2”, and lower left lip cancer “T1”, N2cM0.

**Fig. 2:**
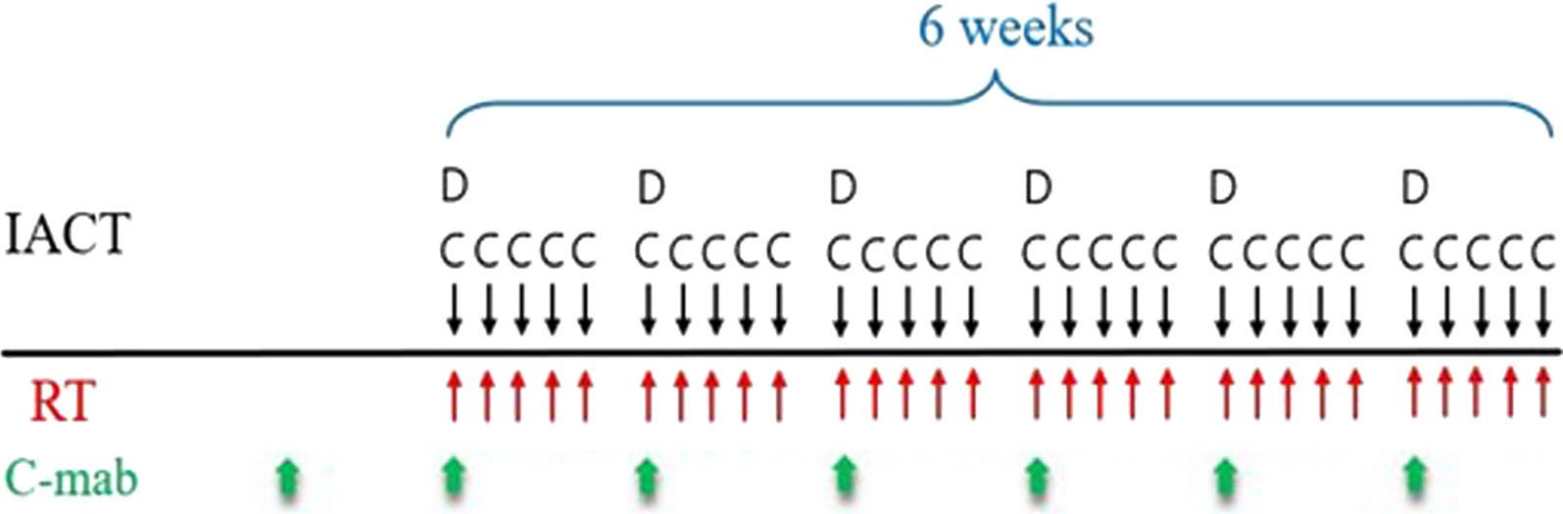
Treatment schedule of IACRT combined with systemic cetuximab administration. IACT: intra-arterial chemotherapy, Cisplatin (C): 5 mg/m^2^/day (Total: 150 mg/m^2^/6 weeks), Docetaxel (D): 10 mg/m^2^/week (Total: 60 mg/m^2^/6 weeks), RT: Radiotherapy, 2 Gy/day (Total: 60 Gy), C-mab: cetuximab, initial dose at 400 mg/m^2^, followed by the dose at 250 mg/m^2^ (Total: 1900 mg/m^2^/7 weeks)

**Fig. 3:**
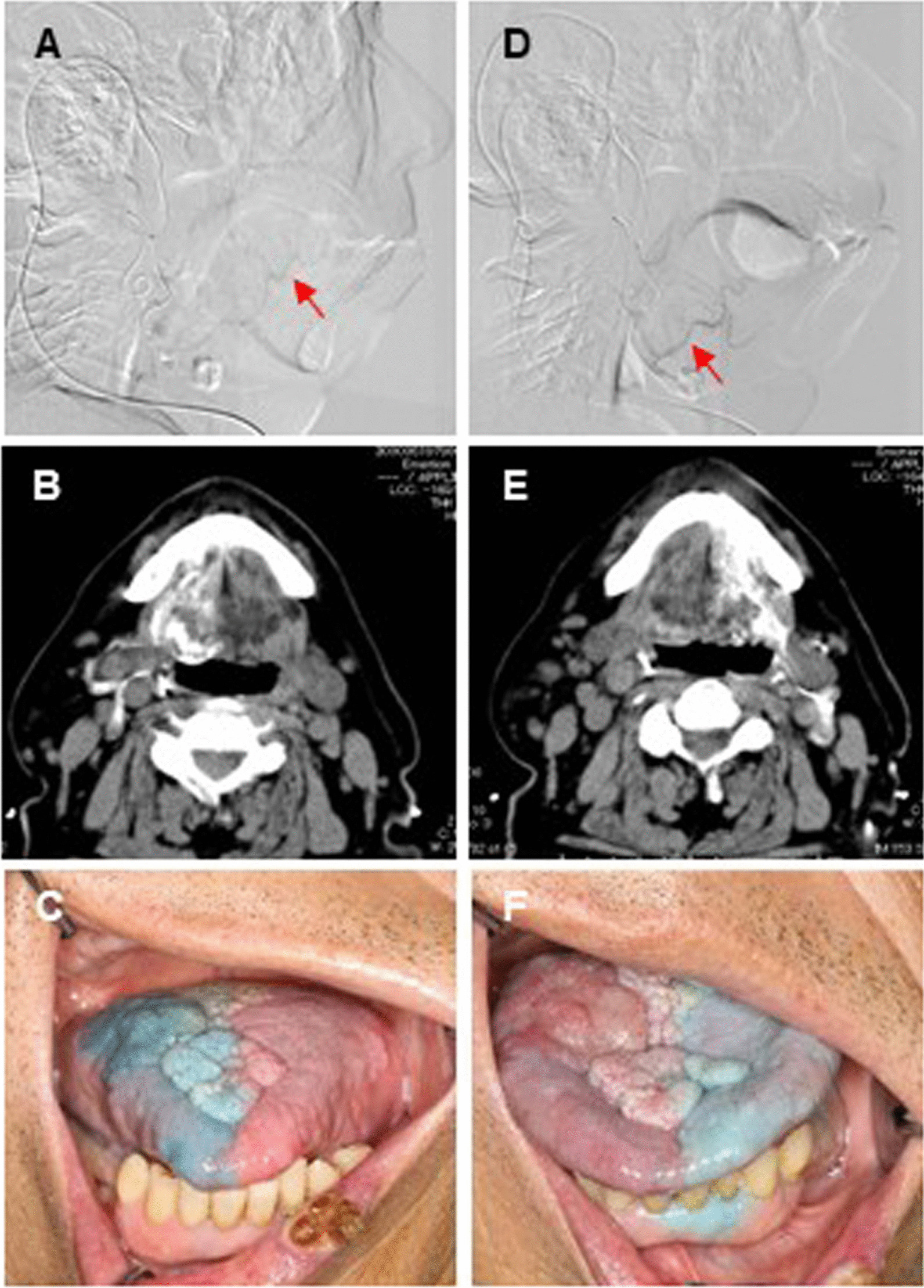
Representative images of DSA, angio CT and indigotindisulfonate-staining. Two catheters were superselectively inserted into the lingual arteries (arrows) via the superfacial temporal arteries (**A**, **B**: flow-check digital subtraction angiography (DSA), **C**, **D**: angiographic CT images). EF, photograph representing the flow areas stained by injection of indigotindisulfonate sodium (**A**, **C**, **E**: right, **B**, **D**, **F**: left side)

The patient received IACRT combined with intravenous (IV) administration of cetuximab. Catheterization from the superior temporal artery (STA) was performed as previously reported [9]. Before the treatment, 3-dimensional computed tomography angiography of the carotid artery was performed to identify the tumor-feeding arteries and examine the morphology of the tumor-feeding artery arising from the external carotid artery. A hook-shaped catheter (Medikit Corp., Tokyo, Japan) was superselectively inserted into the target artery under radiographic guidance and fixed to the periauricular skin. When catheterization using a hook-shaped catheter was not stable, we replaced it with a P–U catheter that is flexible, reducing long-lasting damage to blood vessels (Toray Medical Co., Ltd., Tokyo, Japan). The catheters were superselectively inserted into the bilateral lingual arteries (LA) via STA. Due to preventing the reduction of chemo-drug concentration by distribution through several routes, we decided to exclude the infusion of chemo-drug into left facial artery (FA) targeting lower lip cancer. Treatment was performed for 7 weeks as following schedule (Fig. 2): After catheterization, flow-check digital subtraction angiography and angiographic CT were performed to confirm the appropriate catheter placement and enhancement of the feeding areas (Fig. 3A–D). Sodium indigotindisulfonate was utilized weekly as another confirmation of feeding areas via dyeing of the tongue and oral floor (Fig. 3E, F). Cetuximab was weekly IV administered 1 week prior to the initiation of IACRT; the patient received seven times (400 mg/m^2^ as an initial dose, followed by 250 mg/m^2^, in week 2–7, Fig. 2). To minimize the risk of infusion reaction, antihistamine and corticosteroids were premedicated, and the patient was monitored vital signs [10]. Docetaxel (DTX) and cisplatin (CDDP) were injected as a slow bolus over 1 h through the catheter during the irradiation. The dose of DTX was 10 mg/m^2^/week, for a total of 60 mg/m^2^ during the whole treatment course, and that of CDDP was 5 mg/m^2^/day, for a total of 150 mg/m^2^. Sodium thiosulfate, a CDDP neutralizing agent, was also administered intravenously at 1 g/m^2^ immediately after arterial infusion of CDDP. Radiotherapy was performed 5 times per week using 2 Gy per fraction of 6-MV photon beams with a linear accelerator with a total dose of 60 Gy. The irradiation field was set up to cover the primary lesions and the whole neck. After a total dose of 40 Gy was delivered to the initial field, an additional 20 Gy was delivered to the primary tumors and metastatic lymph nodes within the shrunken field [11].

During the treatment, there were some adverse events: grade 3 oral mucositis, grade 3 cheilitis and grade 2 radiation dermatitis, classified according to the National Cancer Institute Common Toxicity Criteria for Adverse Events ver. 4.0. There were no significant hematological and kidney toxicities or major complications, such as cerebral infarction or other neurological disorders observed.

**Fig. 4:**
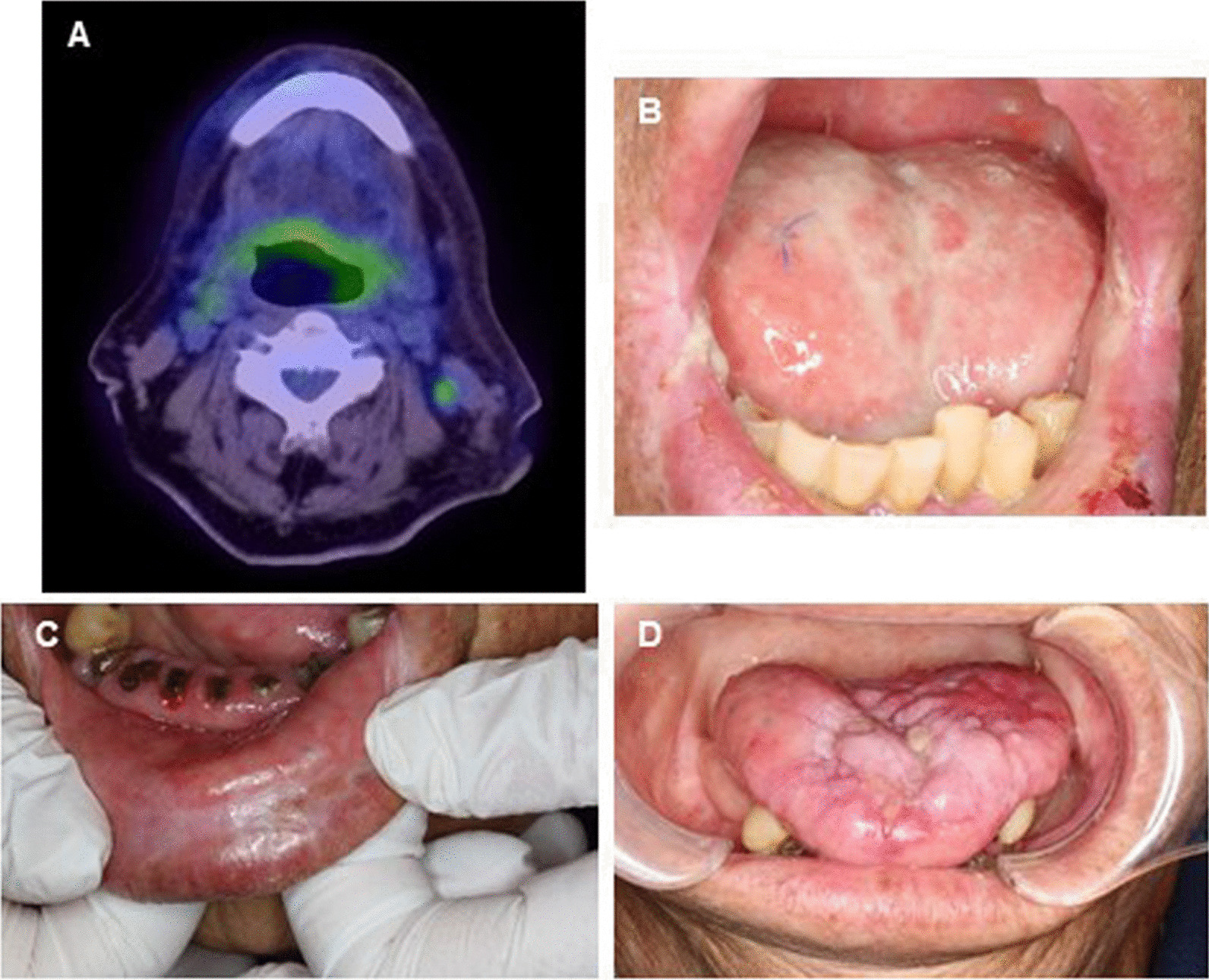
Representative images of synchronous multifocal oral squamous cell carcinomas following completion of treatment. Result of PET/CT one month after the treatment (**A**), local finding one month after the treatment (**B**), and local finding 3 years after the treatment (**C**, **D**)

About 1 month after the completion of initial treatment, no residual local tumors were found by CT and MRI imaging and biopsy, demonstrating that the patient achieved a complete response (Fig. 4A, B). There was a mild FDG accumulation on the left upper deep cervical lymph nodes (Fig. 4A), hence the left modified radical neck dissection was conducted. No viable cancer cells in the dissected lymph nodes were found, resulting in pathological complete response. During the subsequent follow-up period, no evidence of recurrence and metastasis was suggested, and the patient did not show dysarthria and masticatory disturbance for four years so far (Fig. 4C, D).

## Discussion and conclusions

Although multifocal SCC has been thought to be rare in all cancers, those cases are currently increasing with extended life expectancy. Since Billworth et al. reported the occurrence of multiple malignant tumors in 1879, many authors began to report similar cases, making the tumors no longer to be a curiosity [12]. A number of studies have described the multifocal occurrence of SCC in the upper aerodigestive tract [13]. The synchronous multifocal carcinoma is defined by Warren and Gates [14] in 1932, and also by Moertal et al. [15] in 1961, including: (1) all the tumors had to be histologically malignant; (2) all had to be distinct masses separated by normal tissue (at least by 2 cm); (3) the possibility of metastatic tumors had to be excluded histologically. Moreover, synchronous tumors are defined as second neoplasms at the same time or within 6 months of primary lesions [16]. In the present case, three tumors were detected at the initial visit and then diagnosed as SCC simultaneously.

In the treatment of multifocal cancer in the oral cavity, a limited number of clinical studies have been reported. The majority of them reported surgical resection or radiation therapy including various methods, such as external and internal irradiation [17, 18], telecobalt radiation [19], and radiation combined with laser therapy [20]. An extensive surgical resection may cause functional and esthetic disorders and impact patient’s QOL. Therefore, IACRT combined with IV administration of cetuximab was thought to be good indication.

In consideration of the rationale for selecting this regimen, there are pros and cons; IACRT is available to administer high concentrations of anticancer drugs into the tumor directly via feeding arteries, obtaining high efficacy and minimizing the adverse events and functional disorders due to reducing the circulation of chemo-drug systemically. However, this treatment targets locally advanced tumor, it may miss an occasion to be completely cured when the tumor spreads into multiple cervical lymph nodes and/or distant regions. In retrograde superselective IACRT method, catheterization could be performed from not only STA but also occipital artery (OA). When the tumor had 2 or more feeding arteries, catheters were inserted into the 2 arteries via STA and OA or bilaterally. It is different from the occasional cases or antegrade method, we usually place the catheter tip into the first branches of external carotid artery, not further peripheral brunches. Therefore, targeting arteries are mainly LA, FA, and maxillary artery (MA) for the treatment of oral cancer. The chemo-drug distribution is decided according to the area of blood flow from the tumor-feeding arteries. The main feeding artery is typically used for 3–4 weeks, followed by 2–3 weeks usage from collaterally targeting arteries depending on the location of tumor and the number of feeding arteries. In our previous study, the 5 years overall survival rate of stage III and IV oral cancer treated by IACRT (112 cases) was 71.3% (83.1% for stage III and 64.5% for stage IV), confirming high quality of therapeutic effects [5]. Whereas IACRT is distinguished for local control, distant metastasis is still being the serious cause of mortality. To overcome this issue, we have conducted a clinical study in which combination of systemic administration of cetuximab and IACRT to seek its safety and the regulation of cervical lymph node and distant metastasis.

There are other benefits to combine cetuximab with IACRT in this case. In general, IACRT can target local tumor, it also means that locally-advanced tumor needs to utilize several feeding arteries to deliver the anti-cancer drugs. As there is a limitation of predetermined amount of anti-cancer drug in each arterial route, the efficacy could be halved if the route of administration is dispersed in advanced cases and/or multifocal tumor. In the present case, we did not place the catheter into left facial artery to target the lower lip cancer, because both tongue cancers showed T2 and T3 sizes and were required a certain amount of chemo-drug administration. As the size and location of lip tumor are limited, it was thought to be an indication for the treatment of cetuximab combined with radiation [21].

To the best of our knowledge, this is the first report describing the combination of retrograde superselective IACRT and systemic cetuximab for the treatment of synchronous multifocal SCC in the oral cavity. This treatment seems to be promising for the patients with advanced and/or multifocal oral cancer.

## Data Availability

All data generated or analyzed during this study are included in this published article.

## References

[1] Bray F, Ferlay J, Soerjomataram I, Siegel RL, Torre LA, Jemal A (2018). Global cancer statistics 2018: GLOBICAN estimates of incidence and mortality worldwide for 36 cancers in 185 countries. CA Cancer J Clin.

[2] Crestopher PW, Elisabete W, Bernard WS, editors. World Cancer Report 2020: cancer research for cancer prevention. International Agency for Research on Cancer; 2020. p. 310–316.

[3] Ota Y, Aoki T, Karakida K, Yamazaki H, Makuuchi H, Chino O, Miyasaka M (2000). Simultaneous treatment of multiple primary cancers of the oral cavity and other sites. Tokai J Exp Clin Med.

[4] Henning S, Maximilian UJ (2002). Prospective evaluation of quality of life after oncologic surgery for oral cancer. Int J Oral Maxillofac Surg.

[5] Mitsudo K, Koizumi T, Iida M, Toshinori I, Hideyuki N, Senri O, Mitomu K, Makoto H, Izumi K, Masaharu H (2014). Retrograde superselective intra-arterial chemotherapy and daily concurrent radiotherapy for stage III and IV oral cancer: analysis of therapeutic results in 112 cases. Radiother Oncol.

[6] Mitsudo K, Hayashi Y, Minamiyama S, Nobuhide O, Masaki I, Toshinori I, Senri O, Toshiyuki K, Mitomu K, Makoto H (2018). Chemoradiotherapy using retrograde superselective intra-arterial infusion for tongue cancer: analysis of therapeutic results in 118 cases. Oral Oncol.

[7] Naruse T, Yanamoto S, Matsushita Y, Sakamoto Y, Morishita K, Ohba S, Shiraishi T, Yamada S, Asaba I, Umeda M (2016). Cetuximab for the treatment of locally advanced and recurrent/metastatic oral cancer: an investigation of distant metastasis. Mol Clin Oncol.

[8] Bou-Assaly W, Mukherji S (2010). Cetuximab (Erbitux). Am J Neuroradiol.

[9] Tohnai I, Fuwa N, Hayashi Y, Kaneko R, Tomaru Y, Hibino Y, Ueda M (1998). New superselective intra-arterial infusion via superficial temporal artery for cancer of the tongue and tumour tissue platinum concentration after carboplatin (CBDCA) infusion. Oral Oncol.

[10] Doessegger L, Banholzer ML (2015). Clinical development methodology for infusion-related reactions with monoclonal antibodies. Clin Transl Immunol.

[11] Vincent G, Kian A, Wilfried B, Cai G, Marc H, Johannes AL, Anne L, Quynh-Thu L, Philippe M, Chris N (2014). Delineation of the neck node levels for head and neck tumors: a 2013 update. DAHANCA, EORTC, HKNPCSG, NCIC CTG, NCRI RTOG. TROG consensus guidelines. Radiother Oncol.

[12] Burkes EJ (1973). Multiple cancers of the oral cavity. N Carolina Dental J.

[13] Cianfriglia F, DiGregorio DA, Manieri A (1999). Multiple primary tumours in patients with oral squamous cell carcinoma. Oral Oncol.

[14] Warren S, Gate O (1932). Multiple primary malignant tumors. A survey of the literature and a statistical study. Am J Cancer.

[15] Moertel CG, Dockerty MB, Baggenstoss AH (1961). Multiple primary malignant neoplasms: tumours of multicentric origin. Cancer.

[16] Schwartz LH, Ozsahin M, Zhang GN, Touboul E, de Vataire F, Andolenko P, Lacau-Saint-Guily J, Laugier A, Schlienger M (1994). Synchronous and metachronous head and neck. Cancer.

[17] Sato T, Kamata S, Kawabata K, Nigauri T, Mitani H, Yoshimoto S, Yonekawa H, Miura K, Beppu T, Yonekawa H (2003). A study of the treatment of oral multiple primary cancers. Head Neck Cancer.

[18] Okamoto K, Sato K, Uno K, Koike S (1990). Multicentric carcinoma in the oral cavity and pharynx; a report of eight cases. Pract Otol Pract Otol.

[19] Anilkumar LB, Sudarshan R (2016). Multifocal carcinoma of oral cavity: a case report. JKIMSU.

[20] Shionoya K, Okabe S, Matsuki K, Endo T, Matsuki S, Izumo T, Kirita T, Amagasa T (1992). Clinical evaluation of the oral multiple primary cancer. J Jpn Soc Oral Tumor.

[21] James AB, Paul MH, Jordi G, Roger BC, Christopher UJ, Ranjan KS, David R, Jose B, Sharon AS (2010). Radiotherapy plus cetuximab for locoregionally advanced head and neck cancer: 5-year survival data from a phase 3 randomised trial, and relation between cetuximab-induced rash and survival. Lancet Oncol.

